# Engineering *Escherichia coli* to overproduce aromatic amino acids and derived compounds

**DOI:** 10.1186/s12934-014-0126-z

**Published:** 2014-09-09

**Authors:** Alberto Rodriguez, Juan A Martnez, Noem Flores, Adelfo Escalante, Guillermo Gosset, Francisco Bolivar

**Affiliations:** Departamento de Ingeniera Celular y Biocatlisis, Instituto de Biotecnologa Universidad Nacional Autonoma de Mexico (UNAM), Avenida Universidad 2001, Col. Chamilpa, Cuernavaca, 62210 Morelos, Mexico

**Keywords:** Aromatic compounds, Escherichia coli, Metabolic engineering, Systems biotechnology, Synthetic biology, Shikimate pathway, Phenylalanine, Tyrosine, Tryptophan

## Abstract

The production of aromatic amino acids using fermentation processes with recombinant microorganisms can be an advantageous approach to reach their global demands. In addition, a large array of compounds with alimentary and pharmaceutical applications can potentially be synthesized from intermediates of this metabolic pathway. However, contrary to other amino acids and primary metabolites, the artificial channelling of building blocks from central metabolism towards the aromatic amino acid pathway is complicated to achieve in an efficient manner. The length and complex regulation of this pathway have progressively called for the employment of more integral approaches, promoting the merge of complementary tools and techniques in order to surpass metabolic and regulatory bottlenecks. As a result, relevant insights on the subject have been obtained during the last years, especially with genetically modified strains of *Escherichia coli*. By combining metabolic engineering strategies with developments in synthetic biology, systems biology and bioprocess engineering, notable advances were achieved regarding the generation, characterization and optimization of *E. coli* strains for the overproduction of aromatic amino acids, some of their precursors and related compounds. In this paper we review and compare recent successful reports dealing with the modification of metabolic traits to attain these objectives.

**Electronic supplementary material:**

The online version of this article (doi:10.1186/s12934-014-0126-z) contains supplementary material, which is available to authorized users.

## Introduction

**Figure 1:**
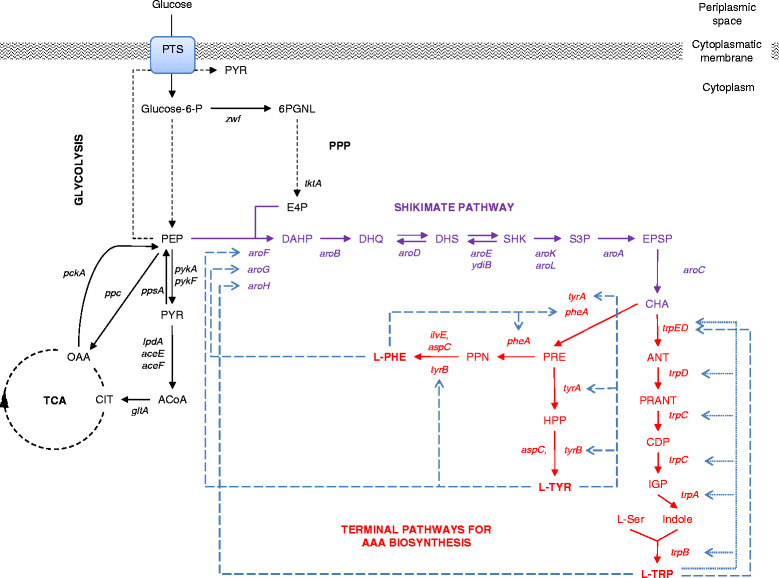
**Schematic representation of the AAA pathway in** ***Escherichia coli*** **including its transcriptional and allosteric regulatory control circuits.** Central carbon metabolism intermediates and genes shown: PPP (pentose phosphate pathway); TCA (tricarboxylic acid cycle); E4P (erythrose-4-P); PGNL (6-phospho D-glucono-1,5-lactone); PEP (phosphoenolpyruvate); PYR (pyruvate); ACoA (acetyl-CoA); CIT (citrate); OAA (oxaloacetate); *zwf* (glucose 6-phosphate-1-dehydrogenase); *tktA* (transketolase I); *pykA, pykF* (pyruvate kinase II and pyruvate kinase I, respectively); *lpdA, aceE,* and *aceF* (coding for PYR dehydrogenase subunits); *gltA* (citrate synthase); *pckA* (PEP carboxykinase); *ppc* (PEP carboxylase); *ppsA* (PEP synthetase). Shikimate pathway intermediates and genes shown: DAHP (3-deoxy-D-*arabino*-heptulosonate-7-phosphate); DHQ (3-dehydroquinate); DHS (3-dehydroshikimate); SHK (shikimate); S3P (SHK-3-phosphate); EPSP (5-enolpyruvyl-shikimate 3-phosphate); CHA (chorismate); *aroF, aroG, aroH* (DAHP synthase AroF, AroG and AroH, respectively); *aroB* (DHQ synthase); *aroD* (DHQ dehydratase); *aroE, ydiB* (SHK dehydrogenase and SHK dehydrogenase / quinate dehydrogenase, respectively); *aroA* (3-phosphoshikimate-1-carboxyvinyltransferase); *aroC* (CHA synthase). Terminal AAA biosynthetic pathways intermediates and genes shown: ANT (anthranilate); PRANT (*N*-(5-phosphoribosyl)-anthranilate); CDP (1-(o-carboxyphenylamino)-1'-deoxyribulose 5'-phosphate); IGP ((1S,2R)-1-C-(indol-3-yl)glycerol 3-phosphate); *trpE*, *trpD* (ANT synthase component I and II, respectively); *trpC* (indole-3-glycerol phosphate synthase / phosphoribosylanthranilate isomerase); *trpA* (indoleglycerol phosphate aldolase); *trpB* (tryptophan synthase); PRE (prephenate); PPN (phenylpyruvate); HPP (4-hydroxyphenylpyruvate); *tyrA, pheA* (TyrA and PheA subunits of the CHA mutase, respectively); *ilvE* (subunit of the branched-chain amino acid aminotransferase)*; aspC* (subunit of aspartate aminotransferase); *tyrB* (tyrosine aminotransferase). Continuous arrows show single enzymatic reactions, black dashed arrows show several enzymatic reactions, long-dashed blue arrows indicate allosteric regulation and dotted blue arrows indicate transcriptional repression. Adapted from EcoCyc database [1].

The aromatic amino acids (AAA), L-tryptophan (L-TRP), L-phenylalanine (L-PHE) and L-tyrosine (L-TYR), are the final products of the aromatic biosynthetic pathway comprising the shikimate (SHK) pathway, which connects central carbon metabolism (CCM) with the biosynthesis of chorismate (CHA), the last common precursor in the terminal branches for AAA biosynthesis (Figure 1) [1],[2]. These pathways are present in bacteria and in several eukaryotic organisms such as ascomycetes fungi, apicomplexans, and plants [3],[4]. The AAA are essential components in the diet of higher animals and humans, hence they are used as dietary supplements (e.g. diet of swine and poultry consisting of grains of corn and soybean is low in L-TRP) and key precursors of industrial and pharmaceutical compounds (e.g. L-PHE is the key ingredient in the synthesis of the artificial sweetener aspartame, whereas L-TYR is an essential dietary component for phenylketonuria patients as the starter material for L-DOPA or melanin production) [5]. The annual worldwide production of amino acids is estimated to be above 4.5 million tons/year, with a market growth for most amino acids of ~10% and higher [6],[7]. Among the aromatic amino acids, L-TRP has a market size of more than 14,000 tons/year [8] and the production of L-PHE exceeds 30,000 tons/year [9].

It is well established that the production of high-valued commodities can be performed cost-efficiently by the rational design, modification and cultivation of a recombinant microorganism. In particular, the development of efficient microbial processes for accumulation of compounds derived from the AAA biosynthetic pathway has not been an easy task for metabolic and bioprocess engineers. For more than 200years, considerable efforts have been directed towards characterizing and purposely overriding the naturally tight metabolic regulation of this pathway. These continued efforts have relied on knowledge obtained from pioneer works on the biosynthesis of aromatic compounds by the groups of B.D. Davis, F. Gibson, C. Yanofsky, A.J. Pittard, K.M. Herrmann and J.W. Frost, among others, whose contributions have been comprehensively reviewed in the past [2],[10]-[12].

Recently, the availability of omics-scale data has allowed significant advances in metabolic reconstruction and modeling, resulting in better strain development [13]. Likewise, the increased use of combinatorial and evolutionary approaches, fueled by a rapid expansion of synthetic molecular tools, opened the possibility for testing novel and large combinations of gene expression systems and genetic backgrounds [14],[15]. Additionally, efforts concerning the optimization of fermentation conditions have succeeded in scaling-up many AAA production processes, while simultaneously providing important feedback on the physiological behavior of engineered strains [16],[17]. However, the availability of operational tools and techniques, as well as the amount of physiological and molecular information, are unevenly distributed among the microorganisms currently used for the production of AAA. These circumstances have contributed to positioning *E. coli* as the organism with most reported success cases and has resulted in a wide array of well-characterized production strains [18],[19].

In this paper we review some notable advances in the generation, characterization and optimization of *E. coli* strains for the overproduction of AAA, some of their important precursors and related compounds. Although these studies were classified in accordance to the main schemes employed for each case, the constant expansion and complementarity of such approaches has encouraged scientists to apply a systems-based perspective [20],[21]. Therefore, recent and representative works on the subject using different strategies were selected and discussed.

### Engineering of the CCM: glucose transport, glycolytic, gluconeogenic, and pentose phosphate pathways

Successful metabolic engineering efforts for the generation of *E. coli* strains that can overproduce AAA include: (i) increasing the availability of the direct precursors phosphoenolpyruvate (PEP) and erythrose-4-phosphate (E4P); (ii) enhancement of the first enzymatic reaction in the SHK pathway to yield 3-deoxy-D-*arabino*-heptulosonate-7-phosphate (DAHP); (iii) improving the carbon flow through the biosynthetic pathway of interest by removal of transcriptional and allosteric regulation; (iv) identifying and relieving rate-limiting enzymatic reactions; (v) preventing loss of carbon flow towards competing pathways; (vi) enhancement of product export; and (vii) prevention of product degradation or re-internalization.

Regarding PEP metabolism, *E. coli* uses the phosphotransferase system (PTS) as the main system for the translocation and phosphorylation of glucose from the periplasmic space to the cytoplasmic environment, consuming one PEP molecule which is converted to pyruvate (PYR) [22],[23]. This reaction yields one molecule of glucose-6-phosphate which is catabolized by the glycolytic pathway, resulting in two PEP molecules (Figure 1). PEP is a precursor feeding several biosynthetic pathways and also participates in ATP generation, either by substrate-level phosphorylation of ADP or indirectly as an acetyl coenzyme-A (ACoA) precursor. When *E. coli* grows in mineral broth containing glucose as the sole carbon source the PTS consumes 50% of the available PEP, whereas the reactions catalyzed by other enzymes such as PEP carboxylase, PYR kinases, UDP-N-acetylglucosamine enolpyruvyl transferase, and DAHP synthases (DAHPS), consume approximately 16%, 15%, 16%, and 3% of remaining PEP, respectively [23],[24]. Therefore, PEP can be converted to PYR by PTS and PYR kinases I and II (coded by *pykF* and *pykA* respectively), and PYR is converted to ACoA by the PYR dehydrogenase multienzyme complex (coded by *aceE, aceF* and *lpd*), a reaction connecting the glycolytic pathway with the tricarboxylic acid cycle (TCA) [1]. Moreover, PEP and PYR are key intermediates of the CCM as they are substrate of at least six enzymes which determine the metabolic fate of these intermediates (biosynthetic/catabolic pathways and glycolytic/gluconeogenic capabilities of the cell): DAHPS isoenzymes (AroF, AroG, and AroH coded by *aroF, aroG* and *aroH*, respectively) [3],[11]; PYR kinases I and II; PEP synthetase (PpsA coded by *ppsA*); PEP carboxylase (Ppc, coded by *ppc*); and PEP carboxykinase (PckA coded by *pckA*) [25] (Figure 1).

Detailed knowledge of these nodes permitted the development of strategies that allowed higher PEP availability for the biosynthesis of aromatic compounds, including the replacement of glucose transport and phosphorylation capabilities of the PTS by alternative enzymes such as the glucose facilitator and glucokinase from *Zymomonas mobilis* (coded by *glf* and *glk*, respectively) [26]-[28], the galactose permease and glucokinase from *E. coli* (coded by *galP* and *glk*, respectively) [29],[30], or the use of an adaptive evolution process to select PTS^−^ derivatives growing at high specific growth rates (*μ*) on glucose [31],[32]. Additionally, high PEP availability has been achieved by modulation of the carbon flux from PEP to the TCA caused by the inactivation of one or both of the PYR kinases [33],[34], as well as improving the recycling of PYR to PEP by a plasmid-encoded copy of PEP synthetase [35]-[37]. The overexpression of *pckA*, in combination with an enhanced carbon flow through the glyoxylate shunt, has also been proposed as a strategy to increase the yield of aromatic compounds [38],[39]. An alternative approach to increase PEP is the attenuation of CsrA, a regulatory protein of carbohydrate metabolism, either by direct gene knockout or by increasing the expression of its negative regulatory RNA, coded by *csrB*[40],[41].

On the other hand, E4P is a metabolite that participates in reversible reactions present in the non-oxidative branch of the pentose phosphate pathway (PPP), as well as a substrate in irreversible reactions that lead to the production of aromatic amino acids or vitamin B_6_[42]. E4P can also be directly produced from sedoheptulose-1,7-bisphosphate in a reaction that is probably favored when the intracellular levels of sedoheptulose-7-phosphate are high [43]. Metabolic engineering reports have shown that a considerable increase in availability of E4P (inferred by the increased production of aromatic compounds and pathway intermediates, such as DAHP) can be achieved by overexpression of genes coding for a transketolase (*tktA)*[35],[44]-[46] or a transaldolase (*talB)*[26],[47]. Additional attempts to increase the carbon flow towards the PPP for enhanced production of aromatic compounds include the use of mutants lacking the enzyme phosphoglucose isomerase [48],[49], the overexpression of enzyme glucose-6-phosphate dehydrogenase [41],[50], or the use of multiple carbon sources, mainly hexoses, pentoses and glycerol [51]-[54]. After an adequate supply of precursors has been established, it is essential to commit this carbon towards the SHK pathway and to remove control points and limiting steps to increase the production of target compounds.

### Deregulation of the AAA pathway: identifying and relieving rate-limiting steps

In *E. coli*, the DAHPS isoenzymes AroG, AroF and AroH contribute to the total DAHPS activity and are subjected to allosteric control by L-PHE, L-TYR and L-TRP, respectively (Figure 1). AroG contributes about 80% of the overall DAHPS activity, AroF about 15%, and the remaining activity corresponds to AroH DAHPS [3],[11]. Both the AroG and AroF isoenzymes are completely inhibited by about 0.10mM of the corresponding amino acids, but AroH is only partially inhibited by L-TRP. Apparent inability of L-TRP to totally inhibit this isoenzyme is proposed to be a mechanism to ensure a sufficient supply of CHA for the biosynthesis of other aromatic compounds when AAA are present in excess in the growth medium [3]. Specific amino acid residues involved in the allosteric sites have been identified by structural analysis of feedback-insensitive mutant enzymes, resulting in the targeted generation of the feedback resistant (fbr) variants AroG^fbr^ and AroF^fbr^[28],[31],[55]. Additionally to allosteric control of DAHPS isoenzymes, their transcriptional expression can be controlled by the *tyr*- and *trp*- repressors complexed with the AAA [3],[11].

Consequently, amplification and deregulation of DAHPS activity is an essential strategy to overproduce aromatic compounds and its precursor SHK. Introduction of plasmid-encoded copies of *aroF*^fbr^ and *aroG*^fbr^ combined with additional plasmid-cloned gene *tktA*, or their chromosomal integrations in gene clusters, have resulted in increased carbon flow from the CCM to the SHK pathway for the production of L-PHE [11],[55],[56], L-TYR [5],[57],[58] and L-TRP [59]-[61]. Positive results were also obtained with the insertion of an *aroG*^fbr^ gene into the chromosome of an L-PHE producing strain while being controlled by a promoter that is active during late cultivation stages, in order to counteract the fall of DAHPS activity in stationary phase [62].

Further increases in carbon flux through the SHK pathway have been attained by the removal of transcriptional and allosteric control points and by relieving limiting enzymatic reactions [2],[11],[19],[23]. The reactions catalyzed by DHQ synthase (encoded by *aroB*) and SHK kinase isoenzymes I and II (encoded by *aroK* and *aroL*, respectively) are considered as rate-limiting [63]-[65]. In addition, the reaction catalyzed by the enzyme quinate/shikimate dehydrogenase (coded by *ydiB*) was also reported as limiting in the development of L-TYR production strains [58]. Either the overexpression of some of these genes by plasmid-cloned copies [28],[66], their co-expression in a modular operon under control of diverse promoters [50],[58],[67], or their expression by chromosomal integration of additional gene copies and promoter engineering by chromosomal evolution [68], have relieved to a great extent these rate-limiting steps typically encountered during the development of SHK and AAA overproducing strains (Table 1). To date, genetically modified *E. coli* strains can overproduce SHK from glucose with yields in the range of 0.08 to 0.420mol SHK / mol glucose under diverse culture conditions [28],[50],[68]-[70]. SHK is a key intermediate of the common biosynthetic aromatic pathway (Figure 1) gaining relevance as the substrate for the chemical synthesis of the drug oseltamivir phosphate, known commercially as Tamiflu, an efficient inhibitor of the surface protein neuraminidase of seasonal influenza, avian influenza H5N1, and human influenza H1N1 viruses [71]-[74].

**Table 1:** **Relevant** ***E. coli*** **strains engineered for the overproduction of compounds derived from the aromatic biosynthetic pathway**

Strain	Relevant characteristics	Main compound produced (titer^a^, and/or yield^b, d^). Relevant culture conditions	References
SP1.1*pts/* pSC6.090B (RB791 derivative)	Δ*ptsHIcrr* Δ*aroK* Δ*aroL serA::aroB* / (plasmid) *aroF* ^fbr^ *tktA,* P_tac_ *aroE serA,* P_tac_ *glf* ^c^ *glk* ^c^	SHK (84, 0.33^b^). 100L fed-batch reactors with glucose, AAA and 150g/L of yeast extract	[28]
AR36 (JM101 derivative)	Δ*ptsHIcrr* Δ*aroK* Δ*aroL* Δ*lacI* Δ*pykF* / (plasmid) P_trc_ *aroB tktA aroG* ^fbr^ *aroE aroD zwf*	SHK (43, 0.42^b^). 10L batch reactors with 1000g/L of glucose and 300g/L of yeast extract	[50]
SA116 (BW25113 derivative)	Δ*aroK* Δ*aroL* P_pps_::P_lacQ1_, P_csrB_::P_lacQ1_ / (chromosome) *aroG* ^fbr^ *tktA aroB aroE*, P_T5_ *ppsA csrB*, 5P_tac_ *tktA nadK*	SHK (3, 0.33^b^). Medium supplemented with 100g/L of glucose, 10g/L of peptone and 10g/L of proline	[68]
W14/pR15BABKG (W3110 derivative)	Δ*crr* Δ*tyrA* / (plasmid) P_R_ *aroG15 tyrB*, P_L_ *pheA* ^fbr^ *ydiB aroK yddG*	L-PHE (47, 0.25^d^). 150L fed-batch reactors with glucose and 10g/L of tyrosine	[132]
FUS4.11/pF81_kan_ (W3110 derivative)	Δ*pheA* Δ*tyrA* Δ*aroF* Δ*lacIZYA* Δ*pykA* Δ*pykF* / (chromosome) P_tac_ *aroF aroB aroL*, (plasmid) P_tac_ *pheA* ^fbr^ *aroF aroB aroL*	L-PHE (13, 0.15^d^). 150L multi-phase fed-batch reactors with glycerol and lactic acid	[131]
BL21 (DE3)	(plasmid) containing the phenylalanine dehydrogenase gene of *Acinetobacter lwoffii*	L-PHE (5, 0.58^d^) 20L batch reactors with 100g/L of glycerol	[130]
MG1655 derivative	(plasmid) P_lac-UV5_ *aroE aroD aroB* ^op^, P_L-tetO1_ *aroG* ^fbr^ *ppsA tktA*, (plasmid) P_lac-UV5_ *tyrB tyrA* ^fbr^ *aroC,* P_trc_ *aroA aroL*	L-TYR (2, 0.44^d^). Shake flask cultures with 50g/L of glucose	[58]
*rpoA* 14^R^ (K-12 derivative)	Δ*pheA* Δ*tyrR* / (chromosome) P_L_ *tyrA* ^fbr^ *aroG* ^fbr^, point mutations in *hisH* and *purF,* (plasmid) *rpoA*	L-TYR (14, 0.12^d^) 20L fed-batch reactors with glucose	[101]
MG1655 derivative	Δ*pheA* Δ*pheL* / (chromosome) P_trc_ *tyrA*	L-TYR (55, 0.30^d^). 2000L fed-batch reactors with glucose	[17]
FB-04/pSV03 (W3110 derivative)	Δ*trpR* Δ*tnaA* Δ*pheA* Δ*tyrA* / (plasmid) *aroF* ^fbr^ *trpE* ^fbr^D	L-TRP (13, 0.10^d^). 30L fed-batch reactors with glucose, 20g/L of L-PHE and 30g/L of L-TYR	[59]
GPT1017 (W3110 derivative)	Δ*trpR* Δ*tnaA* Δ*ptsG* Δ*aroP* Δ*tnaB* Δ*mtr* / (chromosome) swapping of tryptophan attenuator and *trp* promoter by 5CP_tacs_, (plasmid) *aroG* ^fbr^ *trpE* ^fbr^ *tktA*	L-TRP (16). 50L fed-batch reactors with glucose and 10g/L of yeast extract	[80]
TRTH0709/pMEL03 (MG1655 derivative)	Δ*trpR* Δ*tnaA* Δ*pta* Δ*mtr* / (plasmid) *aroG* ^fbr^ *trpE* ^fbr^DCBA *serA*, (plasmid) *tktA ppsA yddG*	L-TRP (49). 300L fed-batch reactors with glucose and 10g/L of yeast extract	[61]
Vio-4 (MG1655 derivative)	Δ*trpR* Δ*tnaA* Δ*sdaA* Δ*lac* Δ*trpL* Δ*gal* Δ*xyl* Δ*fuc* / (chromosome) P_tac_ *aroF aroB aroL tktA serA* ^fbr^ *vioD* ^*f*^ *, trpE* ^fbr^, (plasmid) *vioABCE* ^*g*^	Violacein (0.7). 0.70L fed-batch reactors with arabinose, 120g/L of tryptone and 240g/L of yeast extract	[82]
BKD5 (BW25113 derivative)	Δ*ptsG* Δ*tyrR* Δ*pykA* Δ*pykF* Δ*pheA* / (plasmid) P_lacUV5_ *aroG* ^fbr^ *tyrA* ^fbr^ *aroE*, P_trc_ *ppsA tktA glk*, (plasmid) P_lacUV5_ T7 RNA polymerase, (plasmid) *hpaBC d-ldh* ^h^	Salvianic acid A (7, 0.47^b^). 0.50L fed-batch flasks with glucose and 10g/L of yeast extract	[94]
QH23 (ATCC 31884 derivative)	Δ*pheLA* Δ*tyrA* / (plasmid) P_L lacO1_ *tyrA* ^fbr^ *ppsA tktA aroG* ^fbr^, (plasmid) P_L lacO1_ *tal* ^i, op^ *hpaBC*	Caffeic acid (0.8). Shake flask cultures with 2.50g/L of glucose, 100g/L of glycerol and phenylalanine	[105]
pAD-AG/Δ*tyrR* (BL21 (DE3) derivative)	Δ*tyrR* / (plasmid) *aroG* ^fbr^ *tyrA* ^fbr^, (plasmid) *tal* ^j^	4-coumaric acid (1). Shake flask cultures with 150g/L of glucose	[103]
VH33 ΔtyrR_DOPA (W3110 derivative)	Δ*ptsHIcrr* / (chromosome) P_trc_ *galP*, (plasmid) *tktA* P_lacUV5_ *aroG* ^fbr^, (plasmid) P_trc_ *tyrC* ^c^ *pheA*	L-DOPA (1.5, 0.05^d^). 10L batch reactors with LB and 500g/L glucose	[84]
W3110 *trpD9923*/pS0 *+* pY + pAvnD	(plasmid) P_L-tetO1_ *ydiB aroD aroB aroG* ^fbr^ *ppsA tktA*, (plasmid) P_lacUV5_ *tyrB tyrA* ^fbr^ *aroC aroA aroL*, (plasmid) P_lacUV5_ *HCBT* ^k^ *4CL1* ^l^ *tal* ^i^	Avenanthramide D (27^e^). Shake flask cultures with 100g/L of glucose	[87]

^a^g/L; ^b^mol substrate/mol product; ^c^gene from *Z. mobilis;* ^d^g substrate/g product; ^e^μM; ^f^gene from *J. lividum*; ^g^genes from *C. violaceum*; ^h^gene from *L. pentosus*; ^i^gene from *R. glutinis*; ^j^gene from *S. espanaensis*; ^k^gene from *D. caryophyllus*; ^l^gene from *N. tabacum*; ^op^codon-optimized variant.

In addition to modifications in the SHK pathway, metabolic engineering approaches to overproduce L-TYR typically include alterations in TyrR and/or *trp* regulons. The TyrR regulon comprises diverse essential genes implicated in AAA biosynthesis and transport [1],[75]. TyrR acts as a dual transcriptional activator and repressor; however, the repression mechanism requires the ATP-dependent binding of AAA to the central protein domain. L-TYR is the major effector of TyrR-mediated repression, although some repression occurs with L-PHE as co-repressor for *aroF, aroL, tyrP* (coding for a L-TYR specific permease)*, aroP* (coding for an aromatic amino acid permease) and *aroG* genes, whereas activation does not apparently involve an ATP-dependent binding of aromatic amino acids [1],[2],[5],[11]. Inactivation of TyrR-mediated regulation by deletion of *tyrR* and overexpression of *aroG*^fbr^ and *tyrA*^fbr^, combined with the overexpression of CCM genes (e.g. *ppsA* and *tktA*) and genes of the L-TYR biosynthetic pathway (e.g. *tyrB, aroC, aroA*) have improved the production of L-TYR in diverse *E. coli* strains [5],[11],[58].

Similar results were obtained for L-PHE in resting cells by overexpression of a feedback-resistant or an evolved (ev) CHA mutase/prephenate dehydratase enzymes (coded by *pheA*^fbr^ and *pheA*^ev^, respectively) [55],[76]. The bifunctional enzyme chorismate mutase/prephenate dehydrogenase TyrA, catalyzes the shared first step in L-PHE and L-TYR final biosynthetic pathways (the conversion of CHA to prephenate), as well as the second step in L-TYR biosynthesis (the subsequent NAD^+^-dependent oxidative decarboxylation of prephenate to 4-hydroxyphenylpyruvate) (Figure 1). TyrA catalyzes both reactions in separate domains of the protein and the CHA mutase/prephenate dehydrogenase is feedback-inhibited by L-TYR (up to 95% inhibition of the prephenate dehydrogenase and 45% of the CHA mutase activity) [1],[2]. The bifunctional enzyme CHA mutase/prephenate dehydratase (PheA) also catalyzes the first step in the parallel biosynthetic pathways for L-TYR and L-PHE as well as the second step in L-PHE biosynthetic pathway (prephenate to phenylpyruvate) (Figure 1). The native enzyme is a dimer and each monomer contains a dehydratase active site, a mutase active site and an L-PHE binding site. PheA enzyme is inhibited by L-PHE (up to 90% of the prephenate dehydratase and 55% of the mutase activity) [1],[2]. Feedback-resistant mutants of TyrA and PheA *E. coli* enzymes have been used for the efficient overproduction of L-TYR [11],[17],[57],[67] and L-PHE [55],[76] in combination with some of the previously described alterations in CCM and the SHK pathway (Table 1). An alternative approach to take advantage of the natural feedback-resistant diversity in the TyrA enzyme family was the expression of the TyrC^fbr^ enzyme (cyclohexadienyl dehydrogenase) from *Z. mobilis* and the CHA mutase domain of native PheA from *E. coli*, relieving rate-limiting steps and increasing the carbon flux towards L-TYR [57].

A strategy to minimize carbon loss to competing pathways was exemplified in the CHA node with the construction of L-PHE production strains expressing TyrA enzymes containing tags for increased proteolytic degradation, instead of completely removing the enzyme. The resultant strains have the advantage of not being auxotrophic to L-TYR while displaying a higher L-PHE/L-TYR production ratio than the strain containing the wild-type TyrA [77].

Additional modifications applied in L-TRP overproducers include the overexpression of exporter protein YddG [61],[78],[79], the inactivation of permeases AroP, Mtr and TnaB to avoid re-internalization [61],[79],[80], the deletion of gene *tnaA* coding for a tryptophanase to avoid product degradation [59],[81],[82] and expression of genes included in the tryptophan biosynthetic branch, including a feedback-resistant version of anthranilate synthase, TrpE^fbr^[60],[81].

Variations on the strategies described in this section have also been applied to the production of other valuable compounds derived from the AAA pathway such as phenyllactate, phenylacetate and phenylethanol [83], L-DOPA [84], mandelic acid [85], deoxyviolacein and violacein [82],[86], avenanthramides [87], and resveratrol [88] (Table 2).

**Table 2:** **Proposed applications of high-valued compounds derived from the aromatic pathway and synthesized by engineered** ***E. coli*** **strains**

Compound	Summary of pharmaceutical and industrial applications	References
Shikimate ((3R,4S,5R)-3,4,5-trihydroxycyclohexene-1-carboxylic acid)	Antipyretic, antioxidant, anticoagulant, antithrombotic, anti-inflammatory, and analgesic agent. Has a key role in the synthesis of important pharmacological compounds such as anti-cancer and antibacterial agents, as well as hormones. Substrate in the chemical synthesis of the antiviral Tamiflu.	[72],[74]
Salvianic acid or danshensu (3,4-dihydroxyphenyllactic acid)	A naturally occurring plant polyphenolic acid, considered as a superior antioxidant. Its scavenging activities against free hydroxyl radicals and superoxide anion radicals are higher than vitamin C. Has a variety of other pharmacological effects, including improving cerebral blood flow, inhibiting platelet activation and arterial thrombosis, as well as anti-cancer and anti-inflammatory effects.	[94]
(2*S*)-pinocembrin (5,7-dihydroxyflavanone)	Flavonoid with demonstrated activity decreasing the neurological scores, alleviating brain edema, reducing the permeability of blood brain barrier and alleviating cerebral ischemic injury in the middle cerebral artery occlusion in rats. Has been proposed as a novel therapeutic agent to reduce cerebral ischemia/reperfusion and blood brain injury, useful for its antioxidant and anti-apoptotic effects.	[98]
Caffeic acid (3,4-dihydroxycinnamic acid)	Possesses various pharmacological activities including antioxidant, antitumoral, antiviral, antidepressive and antidiabetic functions.	[104]
Resveratrol (3,4',5-trihydroxystilbene)	Potential therapeutic effects in humans as antioxidant, anti-inflammatory, anticancer, and chemopreventive agent.	[99]
Violacein ((3E)-3-[5-(5-hydroxy-1H-indol-3-yl)-2-oxo-1H-pyrrol-3-ylidene]-1H-indol-2-one) and deoxyviolacein	Activity against herpes simplex virus and pathogenic bacteria such as *Staphylococcus aureus* and *Pseudomonas aeruginosa*. Violacein has shown successful activity against leukemia, lung cancer, human uveal melanoma and lymphoma cells, where it mediates apoptosis. It is also an interesting bio-dye showing attractive color tone and stability.	[82]
PDC (2-pyrone-4,6-dicarboxylic acid)	Proposed as a novel starting material for several useful synthetic polymers such as polyesters and polyamides.	[100]
(*S*)-reticuline ((1S)-1-[(3-hydroxy-4-methoxyphenyl)methyl]-6-methoxy-2-methyl-3,4-dihydro-1H-isoquinolin-7-ol)	Building block for benzylisoquinoline alkaloids, including the analgesic compounds morphine and codeine, as well as the antibacterial agents berberine and palmatine. Useful in the development of novel antimalarial and anticancer drugs.	[97]
Hydroxytyrosol (3,4-dihydroxyphenylethanol)	Powerful antioxidant activity. Potential antitumoral, antiatherogenic, anti-inflammatory and antiplatelet aggregation agent.	[106]
Avenanthramides	Natural hydroxycinnamoyl anthranilates with antioxidant, anti-inflammatory, and antiproliferative effects, considered to contribute to the health benefits of oatmeal consumption. Potential antitumor activities.	[87]
δ-tocotrienol	Vitamin E component naturally produced by photosynthetic organisms. It has shown to induce apoptosis and inhibit proliferation of cancer cells. Possess to some extent neuroprotective, anticancer, and cholesterol lowering properties.	[95],[96]

**Figure 2:**
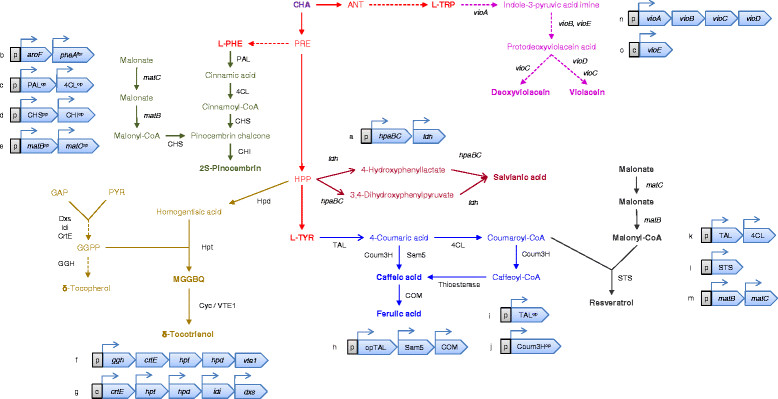
**Biosynthetic pathways for the production of diverse aromatic metabolites by combination of heterologous expression modules with the overproduction of intermediates from SHK- and terminal AAA pathways in** ***Escherichia coli.*** Salvianic acid from HPP: **(a)** *hpaBC* (codes for an endogenous hydroxylase) of *E. coli* and *ldh* (lactate dehydrogenase) of *Lactobacillus pentosus*[94]. 2*S*-pinocembrin from L-PHE and malonyl-CoA: **(b)** *aroF* and *pheA* ^fbr^ of *E. coli*; **(c)** PAL (phenylalanine ammonia lyase) of *Rhodotorula glutinis* and 4CL (4-coumarate-CoA ligase) of *Petroselium crispum*; **(d)** CHS (chalcone synthase) of *Petunia x hybrida* and CHI (chalcone isomerase) of *Medicago sativa;* **(e)** *matB* and *matC* (coding for malonate synthetase and malonate carrier protein) of *Rhizobium trifolii*[98]. δ-tocotrienol **(f)** via MGGBQ (2-methyl-6-geranylgeranyl-benzoquinol) **(g)** from HPP and δ-tocopherol via GGPP (geranylgeranylpyrophosphate): *ggh* (geranylgeranylpyrophosphate reductase) of *Synechocystis sp.*, *crtE* (geranylgeranylpyrophosphate synthase) of *Pantoea ananatis*, *hpt* (homogentisate phytyltransferase) of *Synechocystis sp.*, *hpd* (*p*-hydroxyphenylpyruvate dioxygenase) of *Pseudomonas putida*, *vte1* (tocopherol-cyclase) of *Arabidopsis thaliana*[95], *idi* (isopentenyl-diphosphate isomerase) and *dxs* (1-deoxyxylulose-5-phosphate synthase) of *E. coli*[96]. Caffeic and ferulic acids from L-TYR: **(h)** TAL (tyrosine ammonia lyase) and Sam5 (4-coumarate hydroxylase) of *Saccharothrix espanaensis* and COM (caffeic acid methyltransferase) of *Arabidopsis thaliana*[103]; **(i)** TAL of *R. glutinis* and **(j)** Coum3H (4-coumarate hydroxylase) of *S. espanaensis*[104]. Resveratrol from L-TYR and malonyl-CoA: (k) TAL of *R. glutinis* and 4CL of *P. crispum*; **(l)** STS (stilbene synthase) of *Vitis vinifera*; **(m)** *matB* and *matC* of *R. trifolii*[99]. Deoxyviolacein and violacein from L-TRP: **(n)** *vioABCD* genes of *Chromobacterium violaceum* and **(o)** *vioE* of *Janthinobacterium lividum*[82]. Continuous arrows show unique enzymatic reactions; dashed arrows show several enzymatic reactions. GAP: glyceraldehyde-3-phosphate. c, indicates chromosomal integration. p, indicates plasmid expression module. ^fbr^, feedback resistant gene. ^op^, codon-optimized gene. ↱, promoter.

**Figure 3:**
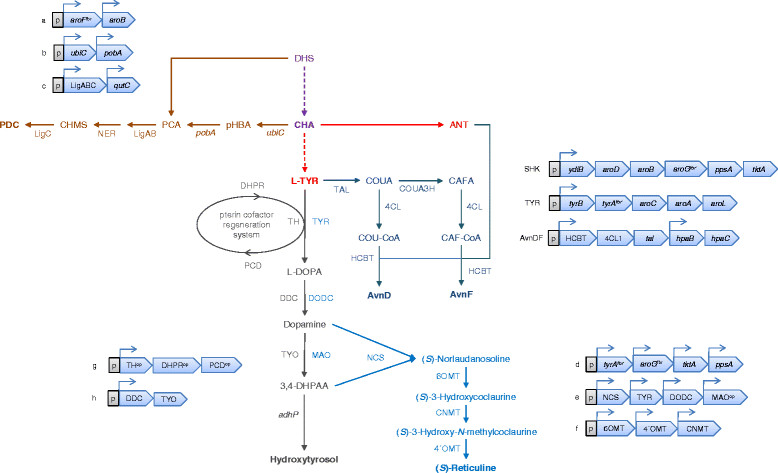
**Biosynthetic pathways for the production of diverse aromatic metabolites by combination of heterologous expression modules with the overproduction of intermediates from SHK- and terminal L-TYR pathways in** ***Escherichia coli.*** PDC (2-pyrone-4,6-dicarboxylic acid) from DHS and CHA: **(a)** *aroF* ^fbr^ and *aroB* of *E. coli;* **(b)** *ubiC* (chorismate pyruvate-lyase) and *pobA* (4-hydroxybenzoate hydroxylase) of *E. coli* and *Pseudomonas putida*, respectively; **(c)** LigAB (protocatechuate 4,5-dioxygenase) and LigC (CHMS dehydrogenase) of Sphingobium sp. SYK-6 and *qutC* (dehydroshikimate dehydratase) of *Emericella* (*Aspergillus*) *nidulans*[100]. (*S*)-Reticuline from L-TYR: **(d)** *tyrA* ^fbr^, *aroG* ^fbr^, *tktA* and *ppsA* of *E. coli*; **(e)** NCS (norcoclaurine synthetase) of *Coptis japonica*, TYR (tyrosinase) of *Streptomyces castaneoglobisporus*, DODC (DOPA decarboxylase) of *Pseudomonas putida* and MAO (monoamine oxidase) of *Micrococcus luteus;* (f) 6OMT (norcoclaurine 6-O-methyltransferase), 4′OMT (3′-hydroxy-N-methylcoclaurine 4′-O-methyltransferase) and CNMT (coclaurine-N-methyltransferase) of *C. japonica*[97]. Hydroxytyrosol from L-TYR via 3,4-DHPAA (3,4-dihydroxyphenylacetaldehyde): **(g)** PCD (pterin-4 alpha-carbinolamine dehydratase) and DHPR (dihydropteridine reductase) of human and TH (tyrosine hydroxylase) of mouse; **(h)** DDC (L-DOPA decarboxylase) of pig and TYO (tyramine oxidase) of *M. luteus*[106]. Avenanthramides AvnD [*N*-(4′-hydroxycinnamoyl)-anthranilic acid] and AvnF [*N*-(3′,4′-dihydroxycinnamoyl)-anthranilic acid] from L-TYR and ANT: AvnDF module, *tal* (tyrosine ammonia-lyase) of *Rhodotorula glutinis*, 4CL1 (4-coumarate-CoA ligase) of *Nicotiana tabacum*, COUA3H (SAM5) (*p*-coumarate 3-hydroxylase) of *Saccharothrix espanesis*, HCBT (hydroxycinnamoyl/benzoyl-CoA/anthranilate N-hydroxycinnamoyl/benzoyltransferase) of *Dianthus caryophyllus* and *hpaBC* (code for an endogenous hydroxylase) of *E. coli*. SHK and TYR modules contain endogenous genes of *E. coli*[56]. PCA (protocatechuate); CHMS (4-carboxy-2-hydroxymuconate-6-semialdehyde); CAFA (caffeate); CAF-CoA (caffeoyl-CoA); COUA (*p*-coumarate); COU-CoA (*p*-coumaroyl-CoA); *adhP* (alcohol dehydrogenase of *E. coli*). p, indicates plasmid expression module. ^fbr^, feedback resistant gene. ^op^, codon-optimized gene. ↱, promoter. NER, Non-enzymatic reaction.

Recombinant pathways are presented in Figure 2 and Figure 3.

### Increasing the genetic engineering repertoire: development and application of synthetic biology strategies and techniques

The field of synthetic biology has been continuously evolving and it is now acknowledged that this discipline is primarily concerned with the design and characterization of biological parts [89],[90]. Indeed, modular and predictable parts find many applications in the modification of cellular metabolism, whether these alterations are direct (modulation of the expression and function of enzymes comprised in metabolic pathways) or indirect (rewiring and repositioning of sensing components and cellular effectors). In this sense, the powerful recent advances in synthesis and assembly of macromolecules have changed the way to approach challenges in metabolic engineering. This has helped to generate a degree of biological diversity and reprogramming not previously reached with traditional biological controllers, promoting the merging of rational and combinatorial approaches to direct cellular design [91],[92].

The aromatic biosynthetic pathway in *E. coli* was no exception to this paradigm shift, resulting in notable accomplishments over the last years. It is worth noting that even when the upregulation of a few genes can increase the carbon flux from CCM towards the aromatic biosynthetic pathway, the outcome is importantly influenced by a variety of factors, such as the combination of expression modules, genetic background and cultivation conditions. It is therefore ideal to design experiments to obtain a characterization of the contribution of each factor to the phenotype. Illustrative examples on this subject include the assessment of differences in the production of L-TYR by overexpressing various sets of genes in a stepwise approach (Table 1) [58],[93].

The generation of synthetic parts in a faster, cheaper and more targeted way has also enabled metabolic engineers to reach unprecedented biochemical diversity, exemplified by the production of plant compounds using precursors present in the aromatic biosynthetic pathway in *E. coli*. In this way, combinations of simultaneous transcriptional modules and genetic platforms have resulted in strains with the ability to produce attractive compounds such as salvianic acid A [94], δ-tocotrienol and its intermediate 2-methyl-6-geranylgeranyl-benzoquinol [95],[96] (Figure 2) and (*S*)-reticuline [97] (Figure 3).

Aside from the product titers reached so far, these approaches are appealing because the systematic evaluation of conditions permits a more precise identification of targets for future improvement. In this respect, more structurally complex compounds can be produced by the optimization of expression parameters, for example when approaching problems with the heterologous insertion of genes and pathways into *E. coli*. One successful case concerning a systematic analysis of heterologous expression is the production of (2*S*)-pinocembrin from glucose as the only carbon source [98] (Figure 2). In this report, the authors assembled gene expression modules, including genes from the SHK pathway as well as heterologous sources. With this arrangement it was possible to accumulate up to 400mg/L of (2*S*)-pinocembrin, even when using four plasmids and enzymes with naturally low catalytic efficiencies. The same system was used to evaluate the capabilities for resveratrol production after slight modifications were introduced to increase malonyl-CoA and L-TYR availability. This work revealed large variations in the concentration of produced metabolites with respect to small variations in the genetic constructs [99] (Figure 2). In a similar approach, the introduction of a foreign pathway succeeded in deviating carbon flow from 3-dehydroshikimate towards the synthesis of 2-pyrone-4,6-dicarboxylic acid (PDC) (Figure 3). Strains overexpressing six different genes from three different plasmids were able to produce the desired compound with a 17.3% yield from glucose [100].

It is also interesting to consider other strategies to generate and screen metabolic diversity, such as modifications of the global transcription machinery coupled to high-throughput screening for metabolite production [101],[102]. By merging these approaches with combinatorial techniques for gene overexpression, a 114% increase in L-TYR production from a previously engineered strain was reported [101].

Strains with the capability to overproduce L-TYR from simple carbon sources have been used as a backbone for production of more structurally complex compounds. For example, the construction of codon-optimized heterologous gene clusters with a wide span of strengths in promoter and ribosome binding sequences (RBS) has allowed the generation of *E. coli* strains capable of producing phenylpropanoic acids such as caffeic acid, coumaric acid and ferulic acid [103]-[105] (Figure 2), as well as hydroxycinnamoyl anthranilates [87] and other derivatives, such as hydroxytyrosol [106] (Figure 3). Another combinatorial technique applied in the generation and isolation of strains with an increased production of indigo (a compound that can be obtained from the L-TRP biosynthetic intermediate indole) is coselection MAGE (multiplex automated genome engineering). This method relies on a cyclical oligo-mediated allelic replacement to modify genomic targets [107] that was later improved by linking the process with the recovery of an inactivated selection marker, enhancing the size and efficiency of insertions [108]. With this approach, the authors were able to insert T7 promoters upstream of 12 genes or operons associated with the AAA pathway in a strain modified to produce indigo and recovered 80 unique derivatives with variable promoter insertions. As a result, it was possible to identify strains with more than a fourfold improvement in indigo production over the ancestor strain, as well as synergetic interactions of expressed genes [108].

The application of synthetic RNA devices with the goal of increasing AAA production in *E. coli* has recently attracted attention. In particular, artificial riboswitches coupling the binding of L-TRP to growth under a selective pressure have been constructed and tested *in vivo*. By modulating the expression of gene *aroG* under this scheme, strains with superior capabilities for L-TRP production could be linked to the increased growth rates after rounds of selective improvement [109]. In another report, a synthetic sRNA library was constructed for targeted gene expression silencing. The authors demonstrated the applicability of this approach in the production of L-TYR with the plasmid-based expression of genes *ppsA*, *tktA*, *aroF*, *aroK*, *tyrC*, *aroG* and *tyrA*, and the simultaneous silencing of genes *tyrR*, *csrA*, *pgi* and *ppc* in several *E. coli* strains. With an easily transferrable gene-regulation platform, the combination of expression levels and genetic backgrounds led to the selection of a strain that can accumulate up to 21.90g/L of L-TYR in high-density cultures [110].

### Integration and application of data: systems-based approaches to the production of AAA

Even with the relative success obtained so far regarding the overproduction of aromatic compounds in *E. coli*, insights into the global metabolic state of engineered strains under production conditions are still scarce. Moreover, the effects of targeted strain modifications are typically underestimated, since they do not always result in significant differences in cell growth or production of specific metabolites. Combination of techniques such as genomics, transcriptomics, proteomics, metabolomics and fluxomics can unravel the particular cellular state during a defined condition by providing snapshots of different levels of metabolism [111]. However, in order to turn this information into knowledge of new potential engineering targets, adequate comparisons must be established. Since it is not trivial to define the type and extent of data to be extracted and compared, systems biology approaches are needed to manage holistic information at different levels of cellular functions [112],[113].

Although the systematic integration of -omics approaches have been applied to characterize and reverse engineer bacterial strains producing several amino acids [6],[114]-[117], there are still relatively few reports on the use of these techniques with AAA overproducers. For example, one study reports the effect of inactivating genes coding for PEP-consuming enzymes (PTS, PykF and PykA) over the flux distributions in the central carbon metabolism as an attempt to increase the availability of this AAA precursor [33]. The net result of either inactivation was a flux increase to biomass formation pathways, but several differences on important CCM nodes were also found between all conditions. Furthermore, PTS inactivation revealed a carbon recycling response between PEP and OAA combined with a reduced glycolytic flux. When these strains were transformed with plasmids encoding enzymes to promote the production of L-PHE, a 19-, 14-, and 25- fold increase on the yield of this amino acid was observed for the PTS, PTS^−^*pykA*, and PTS^*−*^*pyk* F mutants, respectively [33].

Targeted proteomics and metabolite profiling analyses are also very valuable to provide feedback about expression systems used in the production of AAA. One report describes such approaches on a collection of L-TYR producing strains with different gene-expression arrays, allowing the authors to identify and improve sub-optimally expressed genes. After a second engineering round of the synthetic expression modules a strain was constructed which can produce L-TYR from glucose with 80% of the theoretical yield, estimated as 0.550g/g in strains with a functional PTS [58]. A related work characterized the impact on SHK pathway enzyme levels resulting from the removal of TyrR regulator, along with the use of a feedback-resistant TyrA and deletion of the *pheA* gene on L-TYR producing strains. The results showed that small changes in protein levels caused by the genetic alterations can have a big impact on metabolite production, as a 250-fold span of L-TYR concentrations were detected [118]. A different work found many proteins differentially expressed as a response to the sole inactivation of the *pykF* gene, including DAHP synthase (AroG), SHK dehydrogenase (AroE), SHK kinase I (AroK), CHA synthase (AroC), prephenate dehydratase (PheA), anthranilate synthase (TrpD, TrpE) and L-TRP synthase (TrpA), as compared to the wild type strain [119].

In another example, transcriptional analysis and whole genome sequencing studies were performed on L-TYR producing strains obtained by combinatorial and targeted approaches, coupled to high-throughput screening, in an attempt to discover the changes that led to higher L-TYR production [101]. The transcriptional analysis revealed upregulation of genes related to acid stress resistance and global reductions in the expression of several pathways such as ribosomal protein and RNA formation, fatty acid elongation, de novo purine/pyrimidine biosynthesis and DNA replication, which imply a cellular shift from proliferation and growth to maintenance and stress survival. Genomic analyses revealed differential single base-pair changes between the studied strains. When these mutations were reintroduced on a parental strain background higher L-TYR production was observed, showing their contributions to the overproduction phenotype. Finally, a reverse engineered strain was constructed, which gave a titer of 9020mg/L and an L-TYR yield on glucose of 0.180g/g on a genetically-defined background [101]. Other works have also characterized the global transcriptional response to the presence of high levels of L-PHE or SHK in simple and complex media [120],[121] or starvation conditions [122], revealing metabolic information that can be used for further improvement of the strains and cultivation conditions.

Along with data obtained by high-throughput systems, modeling of metabolism by mathematical approaches has become an important tool for analyzing cell responses and unravel the metabolic regulation between the cell information/control systems [111]. Moreover, genome-scale models of metabolism have been analyzed by constraint-based approaches [123]. Gene deletion effects over flux distributions have also been studied in order to find the combination that provides the best metabolic performance on a given condition. For example, the deletion impact of 1261 genes was modeled using a reconstruction of biochemical interactions, resulting in 195 genes exerting high impact on flux distributions in various metabolic subsystems [124]. A strategy developed to circumvent the need for kinetic parameters of enzymes present in a metabolic network is ensemble modeling, which uses phenotypic data obtained from overexpression and deletion of enzymes to screen out flux distributions from an initial ensemble of solutions derived from elementary reaction models [125]. This method has been used to model the AAA pathway for DAHP production with data obtained from the overexpression of CCM genes. A subset of flux distributions was found capable of describing the phenotypic characteristics of the strains and rendered information about the kinetic and stoichiometric limitations around PEP and E4P nodes [125]. As more genomic, transcriptomic and proteomic functional interactions continue to be unraveled, similar approaches will become powerful tools to model specific metabolic outcomes related to AAA biosynthesis.

### Bioprocess engineering: optimization of AAA compound production

In order to create economically viable products, the processes developed and tested at laboratory scale have to be adapted to larger operational volumes. Although engineered strains should ideally perform equally in 10L scale as in industrial scales (going from 500010,0000L for fine chemicals to more than 100,0000L for commodity chemicals), a significant reduction in performance as a result of scale-up is often observed [126]. Therefore, it is important to apply strategies to prevent physiological changes caused by heterogeneities of fermentation parameters during scale-up processes with *E. coli*. Stress-mediated cellular responses to chemical and physical factors can negatively impact as much as 60% over the productivity, the biomass and product yields when a strain is exposed to large-scale production conditions [127],[128].

Fed-batch cultivations have been a popular method to produce aromatic compounds since they promote high cellular densities, offer tight control over the *μ* and substrate concentrations, and permit a better management of dissolved oxygen tension (DOT) to prevent the activation of fermentative pathways [16],[129]. In one example, a fed-batch strategy improved violacein production from arabinose (through an expanded pathway from L-TRP) by adjusting the *μ* at 0.0110h^−1^[82]. With this procedure, cellular concentrations with optical density values up to 70 were reached, producing 7100mg/L of violacein and avoiding acetate accumulation in the medium, a known inhibitor of growth and pigment production.

Another work studied the impact of different feeding strategies over the production of L-TRP in a recombinant strain [16]. An increase in the volumetric productivity of this compound was reached by a novel feeding strategy with a highly concentrated glucose solution (8000g/L) after the exhaustion of the initial glucose. By using a combined pseudo-exponential feeding at the exponential phase and a glucose-stat feeding after the exponential phase, an efficient control over the *μ* was achieved (below the acetic acid production threshold), reaching 38.80g/L of L-TRP. This represented an increase of 19.9% due to reduced acetic acid accumulation [16].

Even if feeding strategies can cope to some extent with the problems derived from acetic acid production, a combination of these with genetic modifications has also been tested for the production of aromatic compounds. In a recent report, the effect of inactivating the gene coding for the enzyme phosphotransacetylase (Pta) over the production of L-TRP was assessed. By combining this modification with the use of a DO-stat for controlling inflow rate at a suitable DOT, the authors were able to increase the production of L-TRP and biomass while maintaining the growth rate and reducing the accumulation of acetic acid [129].

Substrate characteristics can also be optimization variables for the production of AAA pathway intermediates. One example is the evaluation of glycerol for L-PHE production [130],[131]. The low cost of glycerol coupled with its higher degree of carbon reduction when compared to other sugars such as glucose, could result in high energy yield per carbon and hence be advantageous for AAA production processes. However, it is important to characterize the influence of fermentation parameters such as DOT, temperature and pH, as well as the availability of substrates, over the growth and product formation rates. In one report, variations in oxygen supply (by changing aeration rates and impeller speeds) were tested over the L-PHE production capabilities of a recombinant strain growing on glycerol [130]. With this approach, a direct correlation between biomass and L-PHE production rates were found at impeller speeds up to 4000rpm, being this the maximum operational value before shear stress starts to diminish strain capabilities. After setting the impeller speed to 4000rpm, aeration optimization resulted in the highest product yield obtained, 0.580g/g, which is 20% higher than the yields obtained before optimization of oxygen supply. Interestingly, the authors report this high yield with a strain in which the only recombinant measure taken is the heterologous expression of a phenylalanine dehydrogenase gene [130]. On the other hand, another group has recently reported the production of 130g/L of L-PHE from glycerol and a yield of 0.150g/g using a multi-phase fed-batch process with a strain containing several genetic modifications [131] (Table 1).

Product characteristics should also be taken into account when developing an efficient bioprocess. For example, L-TYR exhibits low solubility in typical fermentation conditions, triggering its precipitation when saturation is reached. This characteristic would normally be beneficial for a fermentation process, as a precipitated compound can be easily recovered and it is not expected to affect strain physiology and production capabilities. Interestingly, one report described that the L-TYR crystals can stabilize foam, causing operational problems during fermentation [17]. Consequently, this foaming process was studied on 10 and 2000L fed-batch fermentations for L-TYR production to assess the effect of pH, antifoam concentration, cooling rate, L-PHE concentration and seeding level on foam production. It was determined that high concentrations of L-PHE or antifoam, as well as low pH and low seeding, are the preferred conditions to avoid detrimental foaming production. With this approach it was possible to produce L-TYR from glucose with a yield of 0.30g/g and titers as high as 550g/L on a 2000L scale [17]. Moreover, this study revealed important data for the design of an economically feasible process for the production of L-TYR.

Process optimization could also be concerned with an enhancement of the strain ability to withstand high concentrations of aromatic compounds, not only for toxic final products but for harmful intermediates or byproducts, which often accumulate as a consequence of the suboptimal alleviation of control levels in the biosynthetic pathways. This is a commonly-encountered problem with many of the intermediates and final products in the AAA. For example, one group reported the optimization of L-TRP production by modifying the export and import capabilities of a modified strain in order to minimize its intracellular concentration and avoid feedback control by product accumulation [79]. This group constructed a strain featuring the plasmid-based overexpression of the AAA exporter YddG, resulting in a production increase of 12.6% compared to the parental strain on a 300L fermentation. Another example of the successful combination of genetic and fermentation procedures involves the construction of a strain for L-PHE overproduction with a PTS-independent glucose transport and expression of feedback-resistant versions of AroG and PheA. By overexpressing genes *ydiB*, *aroK* and *tyrB* with a temperature-dependent system, as well as *yddG* in a TyrA^−^ background, the authors were able to produce up to 470g/L of L-PHE with a yield of 0.250g/g from glucose in a 150L fed-batch process [132].

Finally, bioprocess design is also an important factor in optimization of the production of aromatic pathway derivatives. The bioconversion of phenylpyruvate (PPN) to L-PHE was studied with an immobilized cell bioprocess [133]. This technique has several advantages such as the ability to reuse the immobilized cells, the capacity to utilize high cell densities and improved stability of the system. For example, a mixed-gel surface composed of k-carrageenan and gelatin, together with the optimization of its composition to enhance the mechanical strength and reduce the toxicity and solidification point was used as biomass carrier for the production of L-PHE [133]. Studies on the effect of pH, temperature, Mg^+2^ and trehalose presence resulted in the implementation of a process showing an improvement of 80% on the L-PHE conversion from PPN after 15 successive batch experiments.

## Conclusions

The present review aims to provide a panorama of the current achievements and newly found goals related to the production of aromatic compounds in *E. coli.* The AAA pathway and the metabolic changes resulting from its deregulation have attracted the interest of metabolic engineers for many years and remain important research targets on several organisms. It is evident that the establishment of efficient bioprocesses on this topic requires the design and implementation of multidisciplinary strategies, taking advantage of the fast-paced developments coming from nearly all biotechnological fields but particularly from those related with information technologies, such as systems and synthetic biology. The works compiled here are a good example of the benefits obtained when new ideas and viewpoints are introduced to an established field in order to cope with long-known problems. From the comparisons presented, it is noticeable that the use of rational and combinatorial approaches powered by the ability to develop complex genetic circuits and high-throughput screenings of new producers has set new trends when dealing with the production of aromatic compounds in *E. coli*. The benefits of the integral application of these technologies can already be observed, not only from the improved production processes for AAA and pathway intermediates with large and established markets, but also with the generation of novel derivative compounds with important pharmaceutical applications.

## Authors contributions

All authors participated in the preparation of this contribution. AR had a major role in writing and editing the manuscript. All authors have read and approved the final version.

## Authors’ original submitted files for images

Below are the links to the authors’ original submitted files for images. Authors’ original file for figure 1 Authors’ original file for figure 2 Authors’ original file for figure 3
