# Supplementary figures and images for: Engineering Escherichia coli to overproduce aromatic amino acids and derived compounds
Source: Microb Cell Fact. 2014 Sep 9;13:126. doi: 10.1186/s12934-014-0126-z (PMC4174253; doi:10.1186/s12934-014-0126-z)

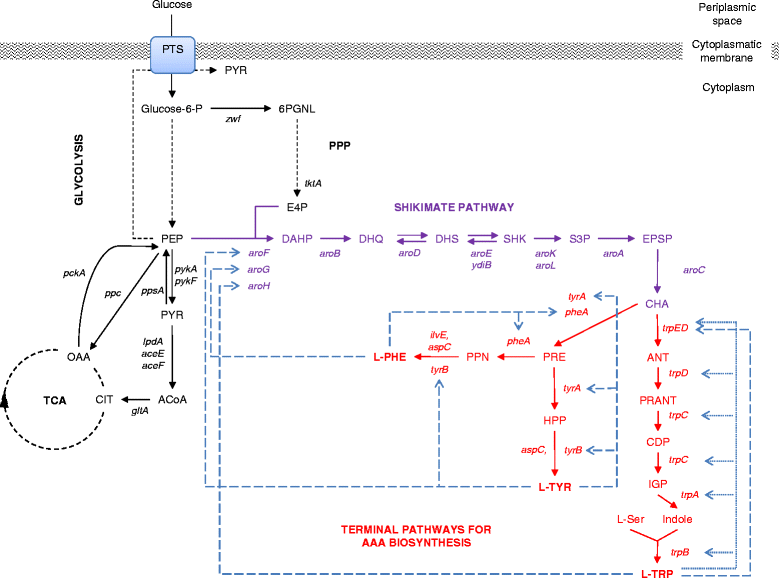

Supplement: Supplementary file 1 — Authors’ original file for figure 1 [file 12934_2014_126_MOESM1_ESM.gif]

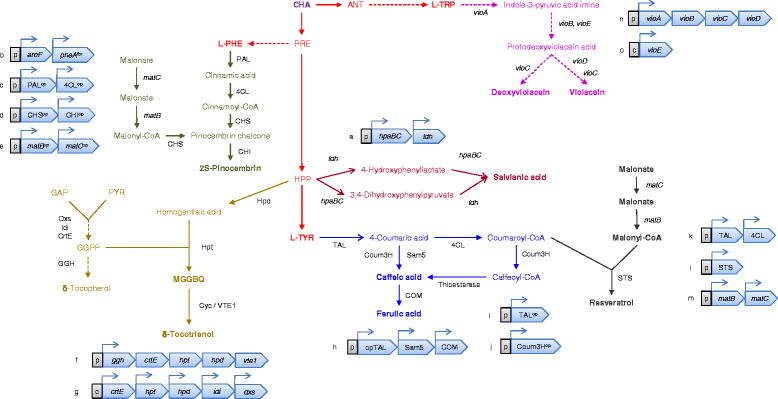

Supplement: Supplementary file 2 — Authors’ original file for figure 2 [file 12934_2014_126_MOESM2_ESM.gif]

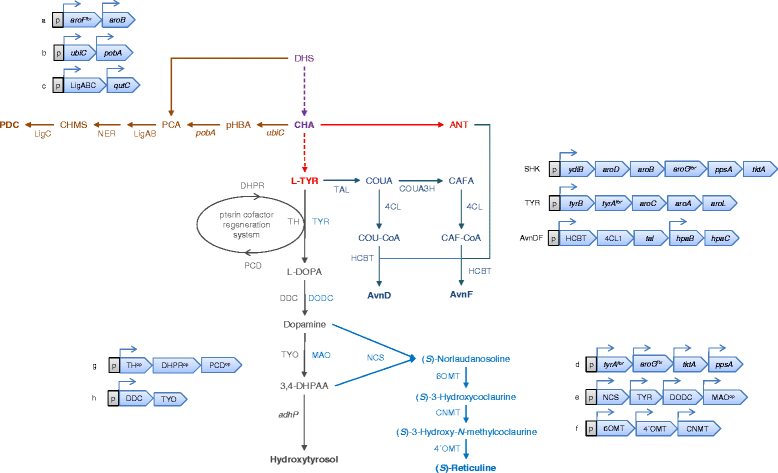

Supplement: Supplementary file 3 — Authors’ original file for figure 3 [file 12934_2014_126_MOESM3_ESM.gif]
